# Non-restrictive cerebral venous dysgenesis in an 11-year-old girl: a case report

**DOI:** 10.1007/s00247-026-06566-6

**Published:** 2026-03-13

**Authors:** Maryam Mozaffari, Rasmus Holmboe Dahl, Malene Landbo Børresen, Tina Duelund Hjortshøj, Goetz Benndorf

**Affiliations:** 1 Department of Radiology, Copenhagen University Hospital – Rigshospitalet, Blegdamsvej 9, Copenhagen Ø, DK 2100 Denmark; 2 Department of Pediatrics, Copenhagen University Hospital – Rigshospitalet, Copenhagen, Denmark; 3 Department of Genetics, Copenhagen University Hospital – Rigshospitalet, Copenhagen, Denmark; 4 Department of Radiology, Baylor College of Medicine, One Baylor Plaza, Houston, TX 77030 USA

**Keywords:** Anatomic variation, Cerebral angiography, Cerebral veins, Cerebrovascular disorders, Chromosome aberrations, Cranial sinuses, Genetic variation, Pediatrics

## Abstract

*Restrictive cerebral venopathy* was recently described in a young patient with cerebral venous ischemia, elevated intracranial pressure, and intracranial calcifications. It was anatomically characterized by extensive formation of tortuous small to medium-sized cortical veins and angiographic absence of the deep venous system. We report similar, albeit not identical, angiographic features in an 11-year-old girl with infantile autism, attention deficit disorder, dyslexia, and camptodactyly. Angiography revealed a venous anomaly characterized by diffuse marked tortuousities involving mainly pial and small cortical veins, partial maldevelopment of the deep venous system, and aplasia of the transverse sinuses. Magnetic resonance imaging showed no signs of venous ischemia. Genetic analyses identified a complex rearrangement involving three chromosomal segments. In conclusion, a unique case of non-restrictive cerebral venous dysgenesis associated with chromotripsis is presented.

## Introduction

Pediatric cerebral venous anomalies comprise a broad category of normal variants and diseases involving the veins and/or dural sinuses. The degree of tortuosity of cerebral veins is usually not considered clinically relevant and may vary from minimal to marked tortuosity [1]. Extensive tortuosity, especially when more focal, is often a consequence of venous hypertension caused by arteriovenous shunts or sinus thrombosis. Thus, the formation of multiple small “corkscrew” veins can be a typical finding in patients with dural arteriovenous fistulas, giving rise to the so-called “pseudo-phlebitic” pattern [2]. Recently, Voronovich et al. [3] reported a novel cerebral venous condition with a generalized pseudo-phlebitic appearance and no signs of arteriovenous shunting in a patient with numerous small- to medium-sized cortical veins showing extensive tortuosities in the absence of larger cortical veins, as well as a missing deep venous system. We report a case of an 11-year-old girl with a similar but somewhat different anomalous venous architecture who underwent cerebral angiography to exclude a suspected arteriovenous shunting. Informed consent was obtained from the patient’s parents for publishing this case report.

## Case report

### Clinical presentation

An 11-year-old girl known with infantile autism, attention deficit disorder, dyslexia, and camptodactyly of both fifth fingers was referred for magnetic resonance imaging (MRI) of the pituitary gland and brain in the context of precocious puberty. A radiograph of her left hand showed a bone age advanced by 3.0 standard deviations (BoneXpert 3.0.3, Visiana, Hørsholm, Denmark). The patient had a history of preterm birth at the 33rd week of gestation due to Kell immunization and received two intrauterine blood transfusions. The neonatal course was uneventful. The patient never had any focal neurological deficits, clinical signs of elevated intracranial pressure, or severe headaches. Her vision and hearing were normal.

### Diagnostic workup

**Fig. 1:**
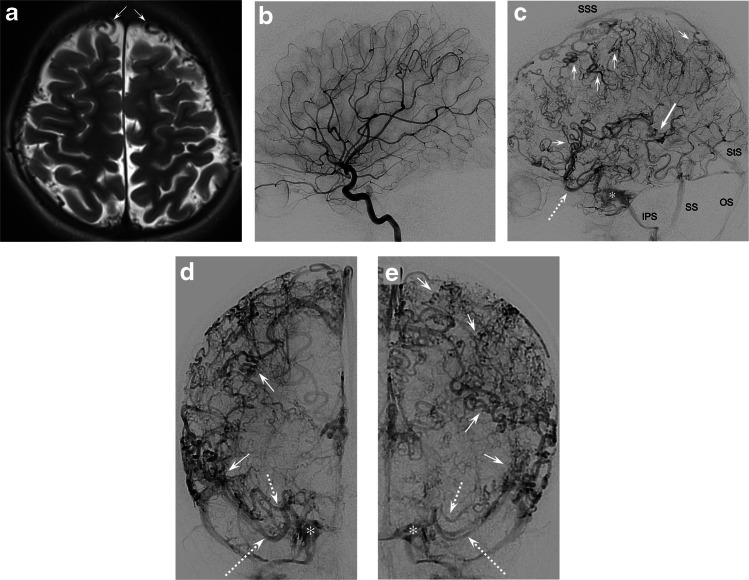
Non-restrictive cerebral venous dysgenesis in an 11-year-old girl with a history of infantile autism, attention deficit disorder, dyslexia, and camptodactyly. **a** Axial unenhanced T2-weighted image shows multiple small tortuous veins in the subarachnoid space above the cerebral hemispheres (*arrows*). **b** 2-dimensional (2-D) digital subtraction angiography (DSA), right internal carotid artery (ICA), mid-arterial phase, lateral: Normal appearance with no signs of arteriovenous shunting. **c** 2-D DSA, right ICA, mid-venous phase, lateral: filling of small superficial cortical veins with marked tortuosities and coilings over the right fronto-parietal and temporal cerebral hemisphere and in the Sylvian territory (*small arrows*). Drainage into the *SSS* and *SS*, and towards the cavernous sinus (CS, *asterisk*) and *IPS* via Sylvian veins (*broken arrow*). Deep venous drainage via tortuous ICV (*long arrow*) into the *StS* and *OS*. **d, e** 2-D DSA, right (**d**) and left (**e**) ICA, mid-venous phase, anteroposterior: extensive tortuosities and coilings of superficial pial and small cortical veins (*small arrows*). Bilateral superficial (*long broken arrow*) and deep (*short broken arrow*) Sylvian veins drain into the CS (*asterisk*). *ICV* internal cerebral vein, *IPS* inferior petrosal sinus, *OS* occipital sinus, *SS* sigmoid sinus, *SSS* superior sagittal sinus, *StS* straight sinus

The initial MRI revealed a slightly enlarged adenohypophysis and multiple small tortuous cortical veins in the basal cisterns, Sylvian fissures, and above both cerebral hemispheres (Fig. 1). The brain parenchyma was unremarkable. A subsequent catheter angiography, including venous three-dimensional (3-D) digital subtraction angiography, showed normal cerebral arterial and capillary phases without arteriovenous shunting. However, during the venous phase, multiple abnormalities were observed in both the superficial and deep cerebral veins as well as the sinuses.

In the *superficial venous system* of both fronto-parietal and temporal regions, numerous tortuous, slightly enlarged pial and small cortical veins were visualized, many of which showed unusual 360-degree coilings. Typical large cortical and bridging veins over the cerebral hemispheres were partly obscured by the dominant opacification of the pial and small cortical veins but appeared rather normal in size and course. *The superficial and deep medullary veins* appeared neither enlarged nor tortuous. In the *deep venous system*, the *subependymal veins* were incompletely developed. The left *internal cerebral vein* showed a normal appearance frontally but its connection to the *vein of Galen* remained angiographically obscure. The right internal cerebral vein had no septal tributaries and appeared very tortuous just before entering the vein of Galen, which appeared patent and of normal caliber. The second and third segments of both *basal veins of Rosenthal* were absent, and the *anterior* and *deep middle cerebral veins* drained towards the *cavernous sinuses*.

**Fig. 2:**
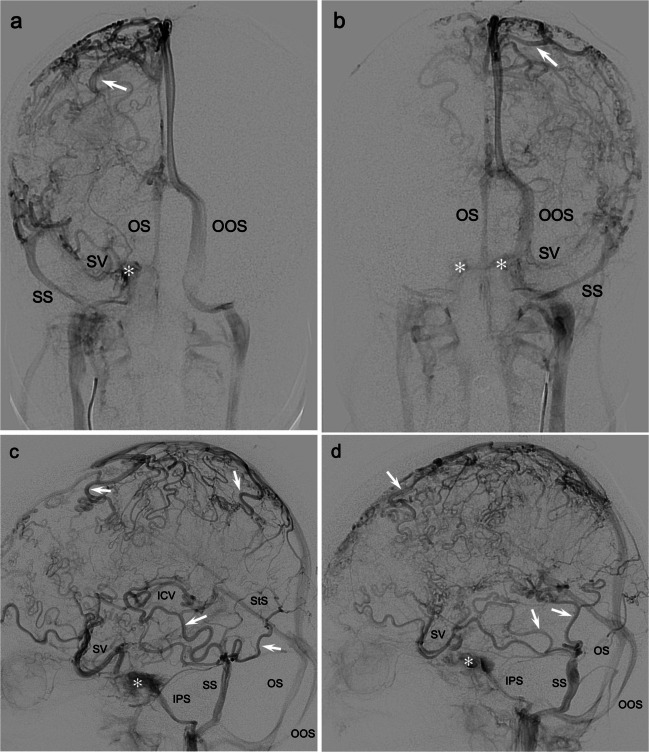
Variations in dural sinus anatomy outlined by 2-dimensional (2-D) digital subtraction angiography (DSA) in an 11-year-old girl with non-restrictive cerebral venous dysgenesis. **a, b** Right (**a**) and left (**b**) internal carotid artery (ICA), late-venous phase, anteroposterior: Abnormal sinus development with bilateral missing transverse sinuses but present *SS* that drains temporo-occipital territories. Small caliber of the frontal superior sagittal sinus (*SSS*) portion with caudal drainage into a left *OOS*. Additional *OS* in the midline that drains the *StS* and vein of Galen system (VoG). The deep *SVs* and cavernous sinuses (*asterisk*) are visible. **c, d** Right (**c**) and left (**d**) ICA, late-venous phase, lateral: bilateral drainage into the *SSs* and *SVs* is shown. The *OSs* are more clearly visible; the larger left *OOS* drains the *SSS* and courses to the left jugular bulb. The smaller midline *OS* drains the *StS* and inferior sagittal sinus and reaches the right jugular bulb. Notably, the larger cortical and bridging veins (*arrows*) are reduced in number in the fronto-parietal regions but are of normal size and appearance. In the temporo-occipital regions, they are moderately tortuous but are normal in number and size. Note the tortuosity of the right *ICV* before it enters the VoG. *ICV* internal cerebral vein, *IPS* inferior petrosal sinus, *OOS* oblique occipital sinus, *OS* occipital sinus, *SS* sigmoid sinus, *StS* straight sinus, *SV* Sylvian veins

In the *cerebral sinus system*, multiple additional abnormalities were found (Fig. 2). The *transverse sinus* was absent on both sides. The *superior sagittal sinus* was hypoplastic in its frontal portion and drained to the left *jugular bulb* via a large left *oblique* *occipital sinus*. The *straight sinus* drained into a second midline occipital sinus, which connected with the right jugular bulb. A prominent *inferior sagittal sinus* drained several smaller, tortuous anterior tributaries on both sides. There were no findings suggesting sinus thrombosis or stenosis. Follow-up MRI after 1.5 months and 2 years showed unchanged findings without signs of cerebral parenchymal calcifications on susceptibility-weighted imaging. This unusual architecture involving part of the superficial and deep venous systems was overall more pronounced on the left side. While the angiographic cerebral circulation time appeared slightly increased due to a prolonged venous phase, the angiogram did not give the impression that the abnormal veins were hemodynamically forced to drain against an outflow restriction. Thus, we speculate that the prolonged venous phase was a result of the extended contrast transit through the markedly elongated and tortuous cerebral veins.

**Fig. 3:**
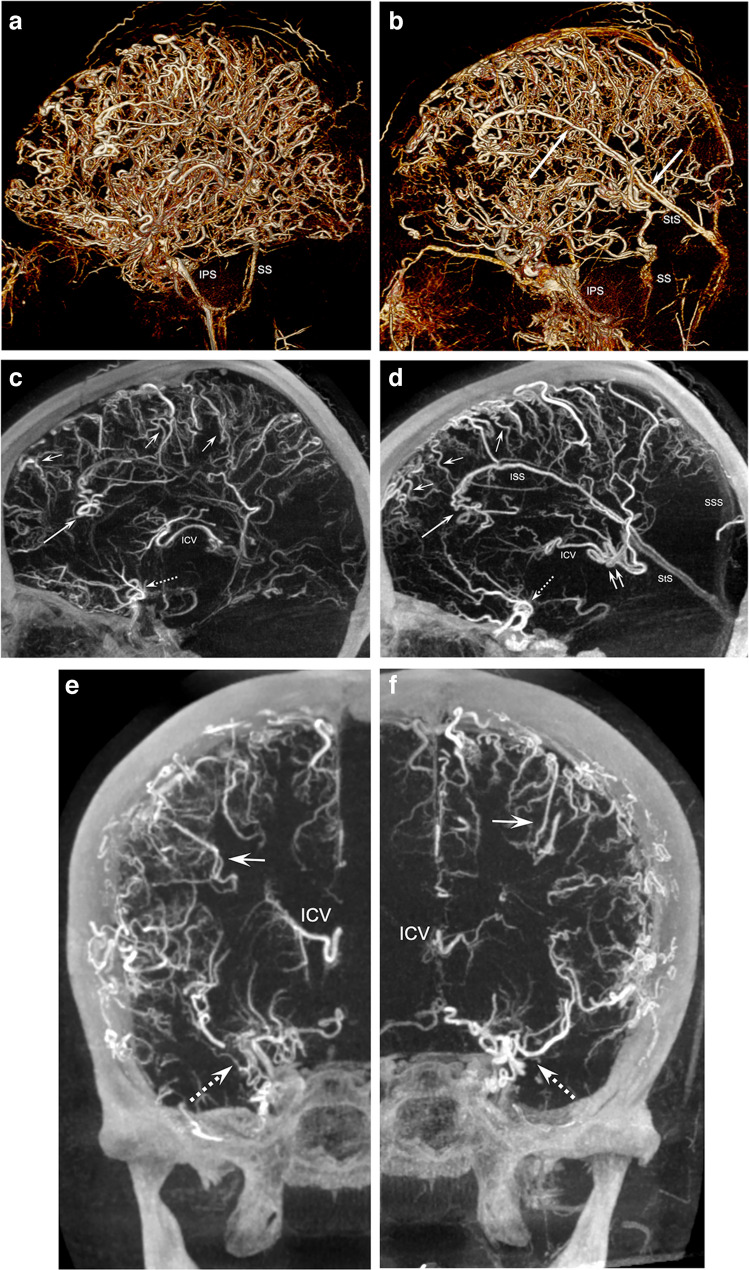

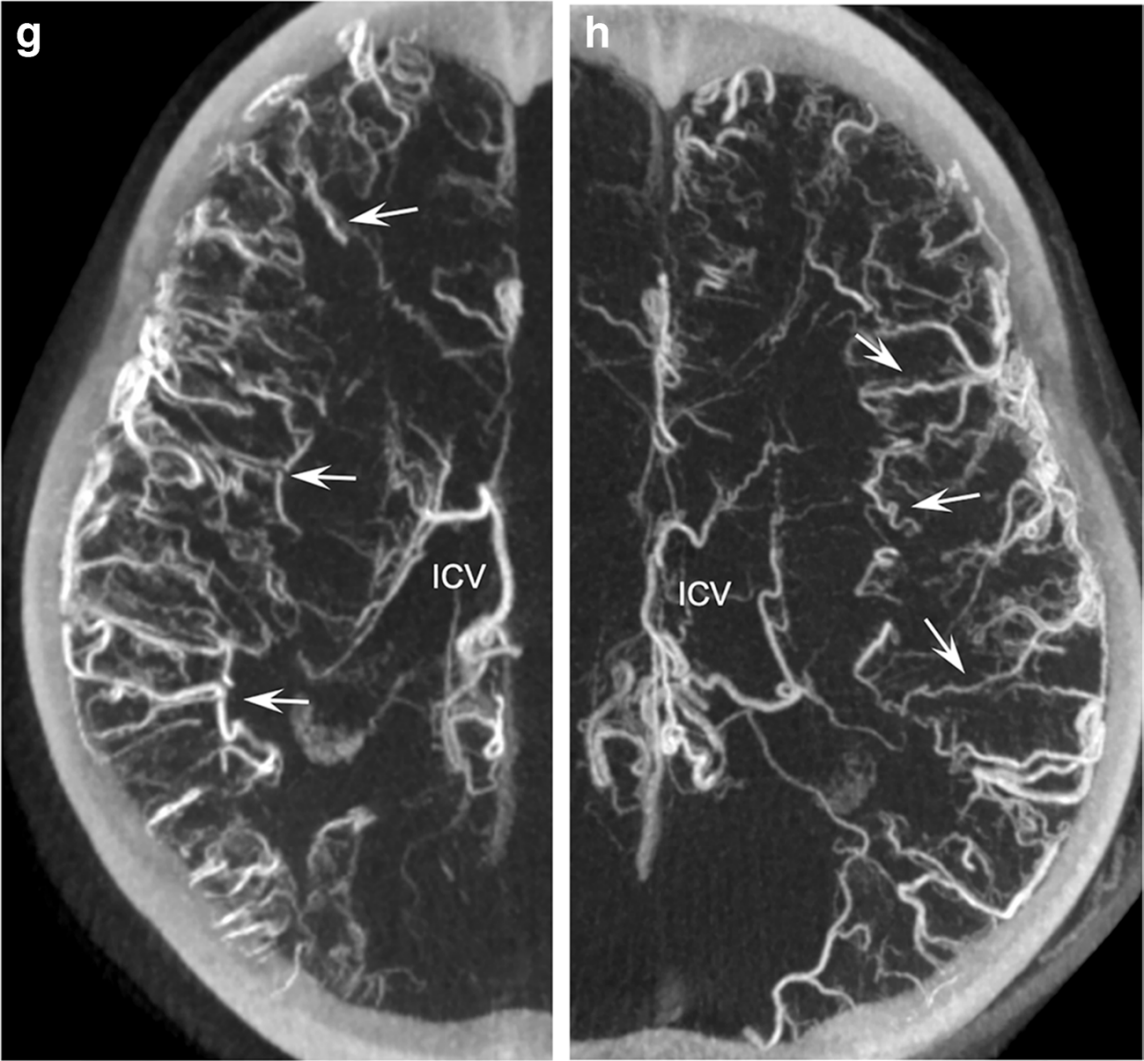
Venous 3-dimensional (3-D) digital subtraction angiography (DSA) in an 11-year-old girl with non-restrictive cerebral venous dysgenesis. **a, b** Volume-rendering technique, right (**a**) and left (**b**) sides, sagittal: the marked tortuosities of pial and small cortical superficial veins on the brain’s lateral (**a**) and medial (**b**) surfaces are more apparent compared to 2-D DSA. Note in (**b**) the dominant visualization of the left *ISS (long arrow*) that drains into the *StS (arrow*). **c, d** Sagittal 20-mm maximum intensity projections, right (**c**) and left (**d**) sides: dilated and tortuous veins near the midline (*small arrows*). The proximal *ISS* drains some additional small tortuous veins (*long arrow*) on both sides (more pronounced filling from the left side), while the distal part appears to travel in parallel to the *StS*. The proximal *ICV* is only partially visualized but also tortuous (*double arrow*) on the left side (not clearly seen on 2-D DSA). **e–h** Coronal 20-mm (**e**, **f**) and axial 25-mm (**g**, **h**) maximum intensity projections, right (**e**, **g**) and left (**f**, **h**) sides. Small pial veins with marked tortuous configurations (*arrows*). The right *ICV* appears more developed than the left. There is no visualization of the bilateral basal veins (2nd and 3rd segments), while there is dominant drainage from the deep Sylvian veins (*broken arrow*) into the cavernous sinus. *ICV* internal cerebral vein, *IPS* inferior petrosal sinus, *ISS* inferior sagittal sinus, *SS* sigmoid sinus, *SSS* superior sagittal sinus, *StS* straight sinus

Some of these abnormal findings, such as the extent of involvement of pial and small cortical veins, were more clearly visualized on maximum intensity projections obtained by venous 3-D digital subtraction angiography (Fig. 3), which were mainly found in the bilateral fronto-parietal regions. The temporo-occipital large cortical veins appeared moderately tortuous but of normal caliber and drained into bilateral sigmoid sinuses. The posterior circulation showed tortuous veins as well, although to a lesser degree and without 360° coilings.

### Genetic workup

Genetic testing was performed by whole genome sequencing and fluorescence in situ hybridization with probes for the terminal p and q arms of the X chromosome. The analysis showed a complex chromosomal anomaly involving the X chromosome and chromosome 10. A 3.4-Mb deletion was found in the pseudoautosomal region 1 of the terminal p arm of the X chromosome. In addition, a 1.9-Mb duplication in the pseudoautosomal region 2 of the terminal q arm of the X chromosome and a 145-kb partial duplication of the *cadherin-related 23 gene* on chromosome 10q22.1 were observed. The duplications in the terminal q arm of the X chromosome and 10q22.1 were fused and translocated to the deleted part of the terminal p arm of the X chromosome. Chromosomal analysis of the parents showed normal chromosomes for both, indicating that the chromosomal rearrangement was de novo in the patient. No other patients with a similar genetic abnormality could be identified, neither in our institutional database nor in international databases such as ClinVar and Database of Genomic Variation and Phenotype in Humans Using Ensembl Resources (DECIPHER). The clinical significance of the genetic finding remains uncertain.

## Discussion

We present a unique case of severely maldeveloped cerebral venous system consisting of extensive tortuosities involving pial and small cortical veins in the absence of arteriovenous shunting and associated with a partial absence or maldeveloped deep venous system. These findings are highly unusual and have been described only in one, somewhat similar, case by Voronovich et al. [3]. The authors observed a 16-year-old boy with developmental delay who presented with seizures, intracranial hypertension, and stroke-like episodes. Angiography showed markedly tortuous small- to medium-sized cortical veins, while the deep venous system, including the vein of Galen and straight sinus, could not be identified. Due to signs of delayed venous outflow on digital subtraction angiography, the presence of intracranial calcifications, and diffusion restriction in the subcortical white matter, suggestive of ischemic changes, the term *restrictive cerebral cortical venopathy* was proposed. Differently, no imaging findings of venous outflow restriction or ischemia were present in our patient. Further, while both basal veins of Rosenthal were absent in their second and third segments, and the left internal cerebral vein was incompletely developed and tortuous, the vein of Galen and straight sinus were patent. On the other hand, both transverse sinuses were absent, and the main deep and cortical venous drainage was directed towards the cavernous sinus and through a normal-sized superior sagittal sinus into two large occipital sinuses.

The most striking finding in our patient were numerous highly unusual 360° coilings of the pial and small cortical veins, a feature not described in the literature so far. The connection from the malformed pial and small cortical veins to the large cortical and bridging veins was difficult to clearly visualize, even on venous 3-D digital subtraction angiography. Whether or not the seemingly reduced number of large cortical veins is directly related to the abnormal appearance of the pial and small cortical veins remains unclear. Similarly, other findings, including the tortuous tributaries to the inferior sagittal sinus, and either tortuous or entirely missing segments of the deep venous system, could not easily be attributed to a reduced number of large cortical veins. We observed some, but not a significant number, of compensatory dilated transcerebral and anastomotic medullary veins as discussed by Voronovich et al. [3].

Overall, the exact causality of the observed abnormalities involving both deep and superficial veins as well as dural sinuses remains difficult to determine. Such cerebral venous dysgenesis without evident outflow restriction would likely be related to a broader developmental failure of the cerebral venous system rather than to an event at a specific stage of embryonic development [3, 4]. In the literature, a few patients with malformed venous systems in the spectrum of *cerebral venous dysgenesis* have been reported; all of them were symptomatic, and none demonstrated the findings detailed in this report [5–7]. Notably, our patient had no seizures or focal neurological symptoms but had a history of infantile autism, attention deficit disorder, and dyslexia. Considering that our patient had no symptoms due to venous ischemia and an otherwise normal brain MRI, it remains debatable whether the presented case is consistent with a true pathological condition or should be regarded as an extremely rare variant of normal.

A complex chromosomal rearrangement involving three chromosomal segments, reflecting a phenomenon called chromotripsis, was found in our patient. Isolated, none of the aberrations is associated with disease, but a positional effect of the duplications cannot be ruled out, as known in other cases with chromotripsis [8]. It is also possible that this genetic finding is purely incidental without clinical consequence. Nevertheless, pediatric neuroradiologists and neurologists should be aware that patients with *cerebral venous dysgenesis* may benefit from genetic testing. More cases and data, including a detailed assessment of the venous anatomy using advanced vascular 3-D imaging, need to be collected to better understand these rare anomalies and their clinical importance.

## Conclusion

We report a case of a potentially novel entity, *non-restrictive cerebral venous dysgenesis*, in a patient with a complex chromosomal rearrangement. This condition consists predominantly of numerous and extensively tortuous and coiled superficial and deep cerebral veins of small size associated with partial maldevelopment and/or absence of the deep veins and dural sinuses. It is possibly related to a failure of normal development of the cerebral venous system. The clinical presentation of *cerebral venous dysgenesis* likely depends on the presence of venous outflow restriction or may be related to associated genetic abnormalities. Whether a causal relationship to an underlying genetic disorder exists in our patient remains uncertain.

## Data Availability

No datasets were generated or analysed during the current study.
